# Engineering Multigenerational Host-Modulated Microbiota against Soilborne Pathogens in Response to Global Climate Change

**DOI:** 10.3390/biology10090865

**Published:** 2021-09-03

**Authors:** Paola Durán, Gonzalo Tortella, Michael J. Sadowsky, Sharon Viscardi, Patricio Javier Barra, Maria de la Luz Mora

**Affiliations:** 1 Scientific and Technological Bioresource Nucleus, Universidad de La Frontera, Temuco 4811230, Chile; patricio.barra@ufrontera.cl (P.J.B.); mariluz.mora@ufrontera.cl (M.d.l.L.M.); 2 Biocontrol Research Laboratory, Universidad de La Frontera, Temuco 4811230, Chile; 3 Centro de Excelencia en Investigación Biotecnológica Aplicada al Medio Ambiente (CIBAMA-BIOREN), Facultad de Ingeniería y Ciencias, Universidad de La Frontera, Temuco 4811230, Chile; gonzalo.tortella@ufrontera.cl; 4 BioTechnology Institute, University of Minnesota, Minneapolis, MN 55108, USA; sadowsky@umn.edu; 5 Núcleo de Investigación en Producción Alimentaria, Facultad de Recursos Naturales, Universidad Católica de Temuco, P.O. Box 15-D, Temuco 4813302, Chile; sviscardi@uct.cl

**Keywords:** suppressive soils, engineering microbiome, biocontrol, food security, sustainability

## Abstract

**Simple Summary:**

In order to face the challenges posed by climate change, scientific research should be directed towards global needs while also keeping into account the need for increased plant productivity. In this sense, our scientific group from the Biocontrol Research Laboratory BIOREN (Temuco, Chile) and our collaborators, have been studying the enormous potential to enhance productivity by using suppressive soils. In this review, we highlight soil-suppressive microbiota as a natural source of biocontrol agents and we propose a strategy to create microbial assemblages, where the plant selects its own inoculants (*when plants “cry for help”*). This approach is based on the selection of specific taxa during the transition from a conducive to a suppressive soil. We hope that this strategy leads to generation of *personalized bioinoculants* to counteract the effects of climate change and increase agricultural sustainability.

**Abstract:**

Crop migration caused by climatic events has favored the emergence of new soilborne diseases, resulting in the colonization of new niches (emerging infectious diseases, EIDs). Soilborne pathogens are extremely persistent in the environment. This is in large part due to their ability to reside in the soil for a long time, even without a host plant, using survival several strategies. In this regard, disease-suppressive soils, characterized by a low disease incidence due to the presence of antagonist microorganisms, can be an excellent opportunity for the study mechanisms of soil-induced immunity, which can be applied in the development of a new generation of bioinoculants. Therefore, here we review the main effects of climate change on crops and pathogens, as well as the potential use of soil-suppressive microbiota as a natural source of biocontrol agents. Based on results of previous studies, we also propose a strategy for the optimization of microbiota assemblages, selected using a host-mediated approach. This process involves an increase in and prevalence of specific taxa during the transition from a conducive to a suppressive soil. This strategy could be used as a model to engineer microbiota assemblages for pathogen suppression, as well as for the reduction of abiotic stresses created due to global climate change.

## 1. Introduction

Due to global climate change, extreme weather events are becoming more frequent, resulting in increased alterations in rainfall events and changes to temperature patterns. Therefore, one main concern of modern agriculture is how plants can tolerate biotic and abiotic stresses without an effect on crop yield, and or reduction of world food security [1]. According to FAO, food security is achieved through four components: availability, access, use, and stability. In this regard, food availability and stability from crops could be at risk due to changes imparted by global climate change. A particular problem in crops is host–pathogen interactions, which influences susceptibility to disease, as well as addressing the distribution of hosts and modifying trade patterns. Moreover, the virulence of pathogens can increase, as well as their geographical expansion, leading to emerging infectious diseases (EIDs) [2]. Most EIDs are caused by pathogens that have increased in incidence, geographical distribution, and/or host range, changed their mode of pathogenesis, or recently evolved, recently discovered, or newly recognized [3,4]. For example, in the case of soilborne pathogens, major disease incidence are expected to cause higher vulnerability of crops due to decreasing resistance to pathogen attacks and migration toward previously uncolonized niches or regions [4,5,6]. An example of EIDs includes *Enterobacter cloacae*, which, although a human pathogen, has been reported to affect plants such as *Allium cepa*, *Morus* sp., *Hylocereus* spp. and *Macadamia integrifolia*, among others [7,8,9,10]. *Xylella fastidiosa* is a plant pathogen that commonly affects grapes (*Vitis vinifera*), but has also been found to attack mulberry leaf [11]. An increased understanding of EIDs is important due to difficulties in predicting, detecting, and diagnosing soilborne pathogens, many of which can survive for many years in the absence of host plants by forming resistant structures such as microsclerotia, sclerotia, chlamydospores, or oospores.

With respect to control, soilborne pathogens have usually been treated using broad-spectrum fungicides and/or fumigants such as methyl bromide, chloropicrin, and metam sodium, among others. However, these have gradually been prohibited because of their negative impacts on human health and the environment [12]. For example, Rivera-Becerril and colleagues evaluated the effects of a pesticide mixture composed of fenhexamid, folpel, and deltamethrin on arbuscular mycorrhizal fungi (AMF) in a vineyard and arable soils, and reported that pesticide application caused a high alteration in the composition of AMF [13]. Usually, chemical control is ineffective because it fails to diffuse efficiently downward into the roots where early protection is essential [14]. Cultural methods such as crop rotation are also applied to reduce disease incidence [15]; however, although effective in disease suppression, crop rotation has the disadvantage that it decreases the opportunity to use plants from similar taxonomic families. For example, *Gaeumannomyces graminis* (Ggt) is a fungus causing take-all disease in cereals, mainly in wheat (*Triticum aestivum*), whose spread is usually prevented by crop rotation. However, this strategy limits the use of susceptible plants belonging to the same family, such as rye (*Secale cereale* L.) or triticale (×Triticosecale, hybrid of wheat and rye). This enhances the probability that take-all disease incidence will increase in key foods, such as cereals, in the coming years, thereby affecting food security [16].

Despite the importance of crop rotation for the control of soilborne pathogens, several reports have shown that the continuous monoculture of a crop can induce specific soil suppressiveness, offering a unique niche harboring specialized microbial communities that can help to suppress disease [17,18,19,20]. In monocultures of soybean, an increased abundance of nematode-trapping fungi and nematode endoparasites has been observed in the rhizosphere and root endosphere of soybean plants, favoring the control of nematode *Heterodera glycines* [20]. In this sense, our group identified six suppressive soils against take-all disease managed by wheat monoculture for more than 10 years [17]. This suppression, also called “specific suppression”, is limited to a particular pathogen and is mediated by one or a few specific microorganisms. Moreover, it is potentially transferable to conducive or non-suppressive soil [14,15,17]. 

**Figure 1:**
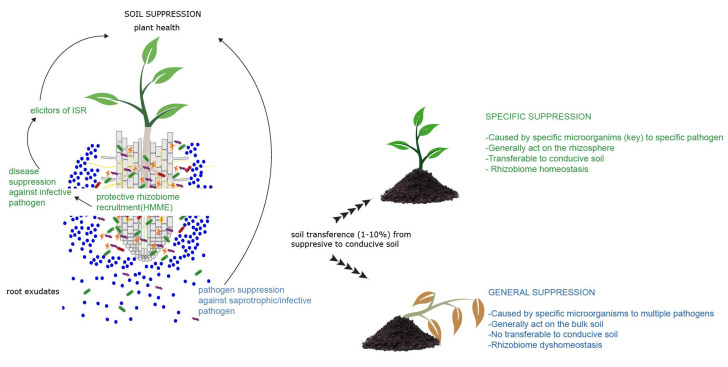
Schematic representation of specific (green letters) and general suppressiveness (blue letters). Specific suppression is limited to a particular pathogen and is mediated by one or a few specific microorganisms. Moreover, it is potentially transferable to conducive or non-suppressive soil. General suppression occurs as a general antagonistic effect exerted by the total soil microbial biomass in the bulk soil against a broad spectrum of pathogens (mainly in saprotrophic phase). General suppression is not transferable between soils.

Specific antagonists can occur anywhere. However, they seem to be most dominant in the soil rhizosphere and influenced by the host plant root (known as the root-associated microbiome or rhizobiome), which is not entirely congruent with the concept of *pathogen suppression* [21]. In this regard, we have not found any significant differences in pathogen DNA concentration between suppressive and conducive soils, but we did demonstrate that suppressive soils have low disease incidence even though the pathogen is present at comparable densities [22]. Similarly, others have reported that higher concentrations of Ggt DNA in roots do not translate into higher disease incidence in plants [23]. In contrast, “general suppression” is based on a general antagonistic effect exerted by the total soil microbial community (biomass) and preexisting soil characteristics against a broad spectrum of soilborne pathogens [18,24]. In general suppression, antagonistic effects occur mainly in the bulk soil, being especially effective against pathogens with a saprotrophic phase (i.e., fungistasis) or influenced by bulk soil chemistry [18]. For example, the important role that soil bacterial populations play on disease suppression, arising from different strategies in organic field management, was revealed via multiple statistical approaches [25]. Therefore, and as illustrated in Figure 1, general suppression is nontransferable between soils, and this is the main characteristic that differentiates it from specific suppression [15,26]. Although it is widely accepted that suppressive functions are in a continuum of responses from general to specific, the former underlies and potentially gives rise to the latter over time, which is conditioned by cropping practices and soil amendments [18]. However, the specific microorganisms as well as their mechanism involved in both general and specific soilborne pathogens’ suppression have only recently been identified, and much remains to be understood.

To date, the human gut is the most studied and characterized microbiome, where microorganisms forming a symbiotic relation are the result of processes of selection and evolution [27]. Considering the importance of gut microorganisms, current scientific efforts are focusing on the manipulation of gut microbiota (engineering microbiome) to enhance human fitness. In fact, microbiota transplantation between humans has gained popularity as a promising therapeutic option for some pathologies [28]. In this context, several authors have considered the root system to be analogous to the human gut, which also has the ability to recruit and select microorganisms [29]. However, unlike soil microbiota, the gut microbiota has a more limited number of genera which can survive the acidic or anoxic conditions prevailing in the gastrointestinal tract. In this sense, the plant-associated microbiota is less known, and it is widely variable depending on the crop, as well as the type of soil and location. Despite the gaps in the knowledge regarding the role that the plant-associated microbiota play, their participation in the growth, root architecture, time of flowering, drought resilience, nutrient uptake, and disease suppression is known [30,31]. Moreover, the root microbiota are not static and can change in a diurnal manner [32]. The microbiota, is defined as the total complex of plant-associated microorganisms, their corresponding interactions, and their genomic machinery [33]. Plants play an active role in recruiting specific microorganisms from bulk soil and therefore modeling their rhizobiome. In fact, different plant species or their genotypes, host-specific associated microbiomes, even grow in the same soil. In addition, from a large portion of microorganisms found in the bulk soil, only a low amount is associated with the plant rhizosphere [33,34,35,36,37,38,39]. These selected microbiota would have an advantage over external microbiota [40], being a “subset” of the plant microbiosphere and acting as reservoir of microorganisms [41,42,43].

In recent years, several fascinating mechanisms involved in specific suppression have attracted scientific interest, specifically with regard to understanding the dynamics in the establishment of particular microbiota in their niche in order to decrease the incidence of a specific soilborne pathogen. Thus, the microbial transition from conducive to specific soil suppression can be understood as natural host-mediated microbiota engineering (HMME), whereby the host indirectly selects microbial communities and triggers host traits that evolve to influence the whole microbiome [44]. This new plant microbiota-manipulating strategy has recently emerged in order to improve positive interactions with plants [40,45], where specific strains consistently associated with a particular host can be used to optimize microbial functions at the individual plant and ecosystem levels [44].

In this review, we discuss the effect of global climate change on emerging infectious diseases (EIDs), the ecological roles played by microorganisms in suppressive soils for infectious diseases, the root-associated microbiome and its role in plant health, and existing methods for natural microbiota engineering, using host-mediated microbiota selection. Moreover, we discuss the use of an interdisciplinary strategy to optimize microbiota assemblages by inducing specific suppression from conducive soil. This model is based on reported antecedents and could be exploited to develop biotechnological strategies based on the use of natural microbiota to fight soilborne pathogens under the imminent climate change scenario.

## 2. Climate Change Effects on Plant Pathogens and Diseases

It is well known that global atmospheric CO_2_ concentration is increasing resulting in climate change. This has brought a series of consequences, such as deep changes in temperature and precipitation patterns [46]. As a consequence of this, the incidence and distribution of plant pathogens is being significantly altered, causing economic losses and crop damages worldwide. Moreover, weather and climate variability can directly influence plant development that lead to higher risk of plant pathogen infection [47]. Regarding airborne pathogens, several efforts have been undertaken in order to elucidate the effect of elevated CO_2_ concentration on plant disease. Interestingly, both beneficial and detrimental effects have been noted, which may be attributed to alterations in stoma structure, stomal conductance, and density under variations induced by climate change, landscape, and plant species [48,49]. This is largely due to the fact that stomata are the entry point for most foliar pathogens. For example, Eastburn and colleagues assessed the influence of elevated CO_2_ concentration (550 ppm) on the ability of three different pathogens (*Septoria glycines*, *Peronospora manshurica* and *F. virguliforme*) to infect soybean [49]. This study showed that disease incidence was decreased in *P. manshurica* and remained without significant variations in *F. virguliforme*. However, when these results were compared to those under a drought period, disease incidence was higher in all cases.

Regarding beneficial effects, the key role of high CO_2_ (1000 and 3000 ppm) in plant defense priming through processes that are linked to redox signaling and metabolism was highlighted, for example, by producing salicylic acid, SA [50]. This was shown to decrease sensitivity to infection against *Botrytis cinerea* and *Pseudomonas syringae* in *Arabidopsis thaliana* and *Phaseolus vulgaris* plants. Similar results were reported where elevated CO_2_ concentration (800 ppm) generally favored SA biosynthesis and signaling, but repressed the jasmonic acid (JA) pathway, which resulted in lower incidence and severity of tobacco mosaic virus (TMV) and *Pseudomonas syringae* in tomato plants [51]. In contrast, the severity of brown spots caused by the fungus *Septoria glycines* increased under elevated CO_2_ (550 ppm) in soybean [49]. Other reports have shown the evolution or appearance of new EIDs to exemplify detrimental effects. For example, *Puccinia graminis* or rust (biotrophic fungi parasitizing cereals) is a significant concern due to the evolution of new virulent races, which can result in ~100% yield loss on wheat under climate change scenarios (warmer climate with lower relative humidity) [52]. A similar tendency was reported for *Puccinia striformis* (yellow rust fungus), where rust fungi increased their virulence and dispersion because of wind. In fact, a foreign incursion of yellow rust fungus in North America, Australia, and Europe has been reported, and this occurred in less than three years [53,54], as seen in Table 1.

**Figure 2:**
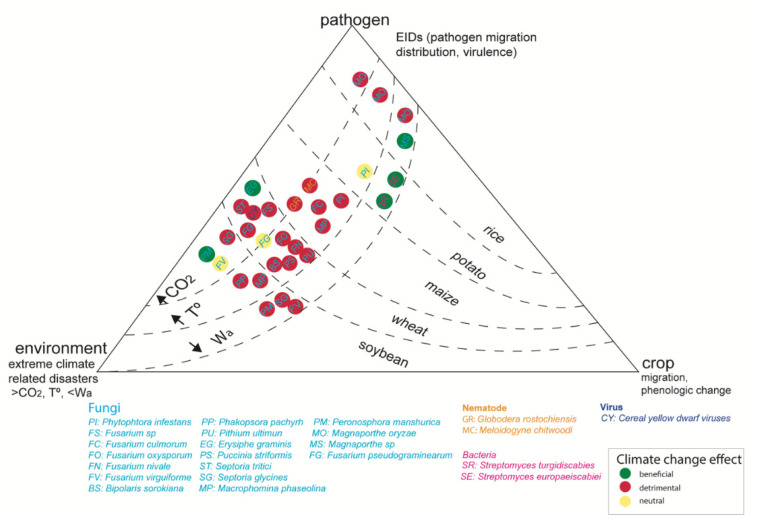
Effect of climate change on key crops (rice, potatoes, cereal, soybean); red: detrimental effect; yellow: neutral or unclear effect; green: desirable effect, under >CO_2_, >T°, <Wa (water availability).

In the case of soilborne pathogens, Chitarra et al. [60] showed that elevated CO_2_ increased the incidence of *Fusarium oxysporum*. Similarly, the authors suggested that a greater frequency of extreme events could be particularly beneficial for *Phytophthora cinnamomi* infections, boosting their density beyond any possible response capacity of susceptible hosts [72]. In contrast, other authors found no significant effects of high CO_2_ (800 ppm) on disease incidence caused by the soilborne pathogens *Rhizoctonia solani* and *Fusarium oxysporum*, compared with ambient CO_2_ conditions (450 ppm, [73]). However, in most studies that evaluated the combined effects of multiple climate change factors on different pathogens, the effects were detrimental. For example, using data from a global field survey and a nine-year field experiment, the importance of soils derived from natural ecosystems as reservoirs for potential fungal plant pathogens was highlighted, denoting temperature as a major environmental factor involved in the global distribution of fungi [74]. Moreover, the authors noted that the proportion of potential plant pathogens will increase in most regions worldwide. Therefore, studies linking disease dynamics under different environmental factors are fundamental to predict the consequences of climate change on plant disease incidence [75]. This is especially important for key crops needed for food security (rice, potatoes, maize, cereal, and soybean). In Figure 2, we summarize the main effects of climate change (increase in CO_2_, temperature, and precipitation) on plant disease affecting key crops.

The impact of climate change on increases in plant pests is also of global concern because a lot of resources are required to control this problem. Zayan [76] indicated that in the US alone, farmers spent more than USD 11 billion for pest control. Additionally, they reported that global climate change could cause some plant pests to undergo from one to five additional lifecycles per season, increasing their ability to overcome plant pest resistance. In the same way, by 2050, the international trade of crops is expected to be seriously affected due to new EIDs which will appear more frequently, as reported by FAO, requiring USD 7 billion per year at that point to deal with this issue. Since the increase in the use of chemical pesticides in crops is unsustainable, it is necessary to use an ecosystem approach involving practices that can guarantee minimal pesticide usage. In this sense, chemical, biological, cultural, and physical methods must be applied together, but in a rational way. Therefore, a new green revolution is required to achieve future food security, where new concepts and approaches are needed to achieve a more sustainable development of agriculture.

## 3. Ecological Roles of Microorganisms from Specific Disease-Suppressive Soils

Soil disease suppression has been described for several soilborne plant pathogens, including bacteria, nematodes, and fungi (Table 2). However, general as well as specific suppression mechanisms have not been fully defined for most suppressive soils. Currently, the development of omics-based approaches has allowed us to understand that specific suppressiveness is caused by specific microorganisms that inhibit the pathogenicity of a specific soilborne pathogen. These microorganisms were recently defined as a key species or core microbiome, in turn driving the microbiome composition and its consequent functionality [77].

Specific suppression could be induced by monoculture practices through the growth of susceptible crops (host) in coexistence with infective pathogens. For example, Ggt-suppressive soil occurrence was examined in 16 locations managed by Indigenous “Mapuche” communities, using monoculture for more than 10 years [17]. Six of these soils were confirmed to be suppressive since they reduced take-all disease in wheat plants. Suppressiveness was lost upon soil sterilization and recovered by adding 1% of natural soil, hence confirming the fact that suppressiveness was closely associated with the soil microbiome community composition. An early study showed that three years of successive wheat cropping could be sufficient for the induction of specific suppression against take-all disease [96]. Later, this was confirmed where it was shown that soils with 3–4 years of wheat monoculture under relatively high pathogen inoculum concentrations were suppressive against take-all disease [23]. In this context, suppressive soils can only occur when three factors occur simultaneously: (i) a monoculture of a susceptible host, (ii) the presence of a pathogen, and (iii) an outbreak of disease [97]. Early reports showed the importance of *Pseudomonas fluorescens* for biological control of take-all disease [98,99]. In this sense, several antagonistic molecules produced by *P. fluorescens*, such as pyolotuerin, phenazine, HCN, pyrrolnitrin, and 2,4-diacetylphloroglucinol, have been reported [100,101]. However, the early interest in *P. fluorescens* as a tool for the biological control of plant pathogens focused on examining it as a single strain, rather than using a set of microorganisms associated with the rhizosphere of plants [101]. This biocontrol ability was attributed to the production of the antibiotic 2,4-diacetylphloroglicinol (2,4-DAPG) [102,103,104]. Other authors showed that specific members of Actinobacteria from a soil suppressive of the fungal root pathogen *R. solani* are able to inhibit fungal growth [105]. Additionally, it has been reported that specific members of the *Burkholderiaceae* family are involved in soil suppressiveness via the production of volatile sulfurous compounds [106]. Additionally, *Streptomyces griseus* was shown to play a fundamental role in the suppressiveness of *Fusarium* wilt of strawberry, caused by *F. oxysporum* f. sp. *fragariae*, via the secretion of lantipeptides which exhibit antibiotic activity [107]. These studies revealed that the rhizobiome provides a first line of defense against the development of soilborne pathogens in periods of monoculture, and in response to high disease incidence or an “outbreak”, such as induced specific suppression [19,108].

## 4. Rhizobiome to the Service of Plant Health, When Plants “Cry for Help”

**Figure 3:**
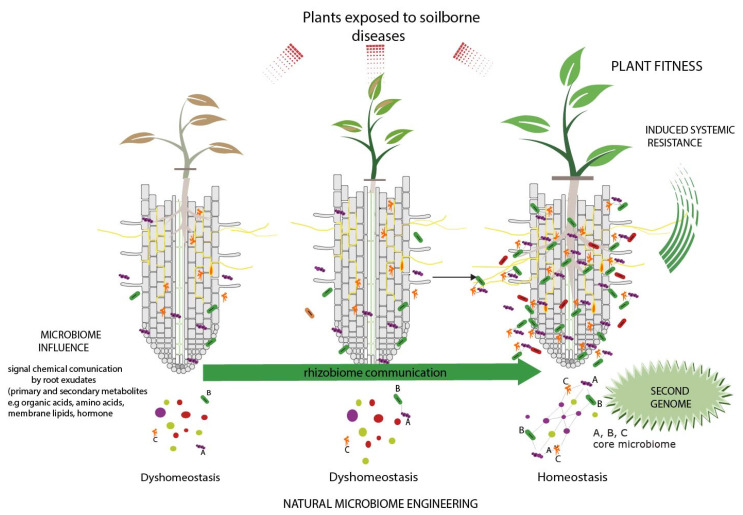
Plants exposed to soilborne disease recruit a specific microbiome (core microbiome or microbial hubs) induced by a chemical signal to induce microbiome homeostasis.

The rhizobiome or rhizosphere microbiome comprises all microorganisms associated with plant roots [109]. Many interactions in the plant–soil–microorganisms complex occur at the rhizobiome level. Indeed, plants can repel or attract (recruit) microbes by using exudates, exerting a significant effect on the general health, or by managing agronomic practices [22,110]. An elegant study showed that *Bacillus cereus* (bacteria implicated in the biocontrol of a wide range of plant pathogens) is able to induce specific components in plant root exudates, which are likely involved in biocontrol processes [110]. For a specific suppression, the “cry for help” concept was suggested, where, at the stage of an outbreak, plants recruit protective microbiota mainly through the exudation of photo-synthetically fixed carbon into the rhizobiome, and favoring endosphere colonization [111,112,113]. The concept of crying for help has been linked to strigolactone (signaling hormones) production, which is involved in the promotion of arbuscular mycorrhizal fungus development and symbiosis establishment under deficient nutrient conditions [114]. Strigolactones have also been observed as a consequence of a herbivore attack, which induced plant volatiles (HIPVs) that contain crucial information for carnivorous insects’ decisions [115]. Recently, four different stages involved in “cry for help” events were proposed and included (Figure 3): (i) root immune responses to belowground and aboveground attackers; (ii) stress-induced changes in root exudation of antimicrobials and semiochemicals (signal molecules); (iii) impacts of root exudates on root-associated and soil-associated microbiota, and (iv) mechanisms by which the root-associated and soil-associated microbiomes suppress pests and diseases [112]. In relation to the first point, several studies have reported that plant roots can generate pattern-triggered immunity (PTI), which contribute to host defense against plant pathogens [116]. Plants have the capacity to recognize potential pathogens via pattern recognition receptors on the cell surface that have the capacity to detect pathogen-associated molecular patterns (PAMPs), such as flagella and lipopolysaccharides, among others, which represent the first-line defense. In this regard, it was reported that typical defense-related genes such as PR-1 and PR-10 are quickly transcribed during the early stages of root infection [117]. The second point is related to second-line defense, which includes the secretion of several molecules that can act as signals for the recruitment of beneficial microorganisms, or that can act directly as antimicrobial compounds [112]. A clear example of this was reported in 2008 [118], where the infection of *Arabidopsis* plants with the pathogen *Pseudomonas syringae* triggered the recruitment of *Bacillus subtilis* to the roots, which act as biocontrol against *P. syringae*. Moreover, the authors reported that the induction of *B. subtilis* by the roots was due to the secretion of malic acid, which is a chemoattractant for this strain. This last point is closely related to the third and fourth stages, since roots have the capacity to affect microbial populations in the rhizosphere either through the recruitment of beneficial bacteria or by actively repressing pathogen proliferation [119]. A good example is the pathogenic fungus Ggt, which can be suppressed in wheat crops via the production of the antibiotic 2,4-diacetylphloroglucinol by fluorescent *Pseudomonas* spp. in the rhizosphere [104].

Currently, specific disease suppressiveness is attributed to the role of functional core microbiota. The core microbiota concept has been defined as the “microbial community that is systematically associated with a specific host plant, where its functions as microbial genes, is essential for the entire holobiont fitness” [120]. In this regard, Trivedi and coworkers tested the potential role of core microbiota, physicochemical properties, and edaphic factors on soil suppression against *F. oxysporum* [121]. The authors identified bacteria belonging to the phyla Actinobacteria, Firmicutes, and Acidobacteria as the major microbial predictors for soil suppressiveness. Similarly, rRNA-based analyses showed that Oxalobacteraceae, Burkholderiaceae, Sphingobacteriaceae, and Sphingomonadaceae were significantly more abundant in the rhizosphere upon *Rhizoctonia solani* invasion, while stress-related bacterial genes representing antifungal activities were specifically upregulated, restricting *Rhizoctonia solani* growth [122]. As discussed above, plants grown in suppressive soils are able to recruit beneficial microorganisms from the bulk soil, and although the entire process of microbial recruitment is not fully elucidated, it is well known that root exudates have a primary role [17,22,84,123,124]. This is possible due to the fact that plants are surrounded by a great diversity and number of microorganisms which can provide several beneficial functions for their plant host [125]. It is thought that the plant rhizosphere contains 10 to 100 times more microbiota than bulk soil, depending on the plant species. This is typically referred to as the rhizosphere effect. Host–microbiome interactions are crucial for plant development. There is ample evidence supporting the fact that rhizosphere microorganisms have a critical role in health, nutrition, productivity, and the overall condition of the plant [22,39,126,127,128,129,130,131,132,133]. The role played by plant growth-promoting (PGP) microorganisms (PGPM) on promoting plant protection has been demonstrated via inoculation with both an individual microorganism and a synthetic mixture of microbiomes [29,79,91,113,128,129,134,135,136]. However, a fundamental challenge for the use of strategies based on microbial inoculation is that synthetic communities often do not persist in soil, and their densities decline rapidly as a result of their competition with indigenous microbiota and adaptation to new environmental conditions [137,138]. Therefore, microbial selection should consider the origin of the microorganisms, their functionality, and their preponderance within the complex microbial community, with core microorganisms being the main targets of search, isolation, and use [139] (Figure 3).

## 5. Core Microbiome—“Few but Good”

Plant microbiota are highly diverse, and these microorganisms are strongly connected with plants, forming complex interactions promoting the productivity and health of the plant in natural environments of the holobiont [140]. However, not all microorganisms play functionally important roles in the host’s biology [141,142]. For example, several studies have reported the presence of a “core microbiota”, a subset of microbial taxa that are consistently associated with a host taxon in a wide range of environments [44,47,123,143]. In fact, many taxa belonging to the core microbiome are likely heritable to subsequent generations and/or fruit postharvest [140,143]. 

The plant core microbiome is composed of key microbial taxa defined as *keystone* or *microbial hubs*, which are critical for plant health based on evolutionary processes that resulted in the selection and enrichment of microbiota carrying genes with essential functions for holobiont fitness [120,144]. Agler [145] defined the term keystone or microbial hubs as specific microorganisms strongly interconnected in the microbial network of plants. However, keystone is differentiated from hub species since the first would be a critical determinant of colonization of widely occurring microbial taxa, and the second would apply to only some specific taxa, not overall [145,146]. A complete summary of keystone taxa in different ecosystems such as grasslands, forests, agricultural lands, Arctic and Antarctic ecosystems, plant-associated microbiome, or phyllosphere, among others, was reported by Banerjee et al. [147]. In this sense, Actinobacteria phylum showed high versatility since that was reported as a keystone taxa in grassland, forest, Antarctic and Artic ecosystems, contaminated soils, aquatic environments, and human gut. For example, the phyla Actinobacteria and Proteobacteria increased positive interactions and strengthened the adaptability of microbiome to grassland degradation [148]. Coincidently, members of phyla Proteobacteria, Actinobacteria, and Bacteroidetes were dominant in both Antarctic vascular plants (*Deschampsia antarctica* and *Colobanthus quitensis*). However, co-occurrence network analyses identified 5 (Microbacteriaceae, Pseudomonaceae, Lactobacillaceae, and Corynebacteriaceae), 23 (Chitinophagaceae and Sphingomonadaceae) and 7 (Rhodospirillaceae) putative keystone taxa present in endosphere, phyllosphere, and rhizosphere, respectively. Thus, niche differentiation in Antarctic vascular plants was evidenced [149]. Another study evaluated the role of a nitrogen-fixing early-colonizer, *Alnus nepalensis*, evidencing that keystone taxa were different at different stages of alder growth [150]. Currently, it is well known that keystone differ to dominant species due to the fact that it might exert their influence on microbiome functioning irrespective of abundance [147], and may be attributable to specific functions such as in the case of plant residue decomposition [151], rhizo deposit stabilization in soil [150], and composted tannery sludge-treated soil [152].

With respect to suppressive soils, a total of 9 and 13 bacterial keystone taxa were identified from suppressive soils against *Ralstonia solanacearum*, and three species belonging to culturable strains of *Pseudomonas* showed high 16S rRNA gene similarity (98.4–100%) with keystone taxa [153]. However, another study showed that the keystone microbiome against *Ralstonia solanacearum* were comprised of the phyla Actinobacteria and Firmicutes and that these microbiota likely plan an important role in diminishing disease incidence [154]. Thus, keystone taxa are highly connected taxa that may exhibit unique and critical roles in organizing the structure of the total microbiome [152].

Microbial hubs are frequently highly connected to other microorganisms inside the network and exert a strong influence on the structure of microbial communities [155]. Thus, hub microbiomes contain more specific microorganisms designed only based on their positions inside a network topology with a specific function, whereas core microorganisms are those with high potential to organize microbiomes in ways that benefit host plants [138].

Microbial hubs and keystones are strongly affected by biotic and abiotic factors that influence the microbial community’s composition [156], and it has been reported that high levels of disturbance and dispersal can interrupt the stabilizing effects of keystone microbial taxa [146]. Therefore, host plants transmit information to the broader microbial network and likely vice versa, and microbial hubs could recruit beneficial organisms or prevent the invasion of pathogens, benefitting the whole system [118,157]. Thus, understanding the plant microbiome’s core functions and the use of synthetic communities as biological control systems provides an opportunity to build a sustainable next-generation agriculture [158]. Finally, microbiome engineering and the identification of specific microorganisms involved in pathogen suppression, such as the core microbiome or microbial hubs, could present a feasible and suitable strategy to solve multiple current agriculture-associated problems in an ecofriendly way, impacting agricultural production, and could serve as a basis for other soilborne pathogens.

## 6. Exploiting Host-Mediated Microbiota Engineering for Protection of Plants against Diseases

Host-mediated microbiota engineering (HMME) is a biological strategy that uses the host phenotype to indirectly select microbiota though cyclic differentiation and propagation [159,160]. HMME is a promising approach to improve host performance by engineering microbial communities for beneficial effects on plant growth and health [44,161]. This strategy enables the selection of a particular microbiome by visualizing phenotypic changes in host plants after several generations of growing in the same place [132]. Thus, modifications in host phenotypes are used to manipulate and select those microbiome functions that impact host fitness [44,162]. While HMME is a novel strategy, it has already been successfully applied to counteract the effect of abiotic stress, such as drought stress [160]. This denotes the great potential of the host as a selective marker to engineer microbiomes that mediate changes in the rhizobiome, improving plant adaptation [160,163]. In the case of biotic stress, specific disease suppression is best exemplified by naturally induced HMME, where plants recruit specific microorganisms from the rhizobiome that can provide plant disease protection [17,19]. The study of [164] assessed distinctive microbiota assembled by maize roots, through host-mediated selection, and studied the role of a simplified synthetic bacterial community composed of even strains from the most dominant phyla: *Enterobacter cloacae*, *Stenotrophomonas maltophilia*, *Ochrobactrum pituitosum*, *Herbaspirillum frisingense*, *P. putida*, *Curtobacterium pusillum*, and *Chryseobacterium indologenes*. These authors found that removing *E. cloacae* led to a complete loss of the community structure, suggesting an important role of keystone taxa in the inhibition of *Fusarium verticillioides*. Later, it was demonstrated in *Arabidopsis thaliana* that, after a disease outbreak caused by *Hyaloperonospora arabidopsidis*, plants were able to assemble protective microbiota in the rhizobiome, inducing systemic resistance [114]. Interestingly, the increased abundance of recruited microorganisms provided protection to the next population of plants growing in the same soil. 

Collectively, these studies highlight the ability of plants to recruit specific microbiota from the rhizobiome with beneficial effects on plant growth and health. Therefore, it is evident that HMME offers a promising novel approach to attain desired host traits. While promising, this approach has only been attempted a few times; nevertheless, it is an extremely powerful technique. In this strategy, microorganisms are forced to co-evolve together with the host plant through successive generations in order to promote a desirable host trait in a manner akin to the natural engineering of plant microbiota. Moreover, the HMME approach applied to both conducive and suppressive states will be very useful to better understand the interactions between plants, microbes, and soils, as well as for the selection of microorganisms directly involved in pathogen suppression. 

## 7. The New Generation of Personalized Bioinoculants by Inducing the Host-Mediated Microbiota Engineering—Perspectives and Future Remarks

An increase in the incidence of plant diseases in agriculture due to climate change effects is imminent. Therefore, novel strategies must be rapidly implemented to ensure food security during times of variable climates. One approach to achieve the goal of plant protection is using HMME taking into account the co-evolutive model of the transition from conducive to suppressive soil. The natural “choice” of “suitable microbiota” by the plant would ensure its success in the face of the imminent changing conditions that are predicted. Moreover, this approach is likely to have much more success than using randomly combined microbiota pools obtained from the rhizosphere of many plant species. The very large number of microorganisms in the rhizosphere coupled with the wide variation in diversity make the construction of microbial pools a daunting task with a low probability of success.

**Figure 4:**
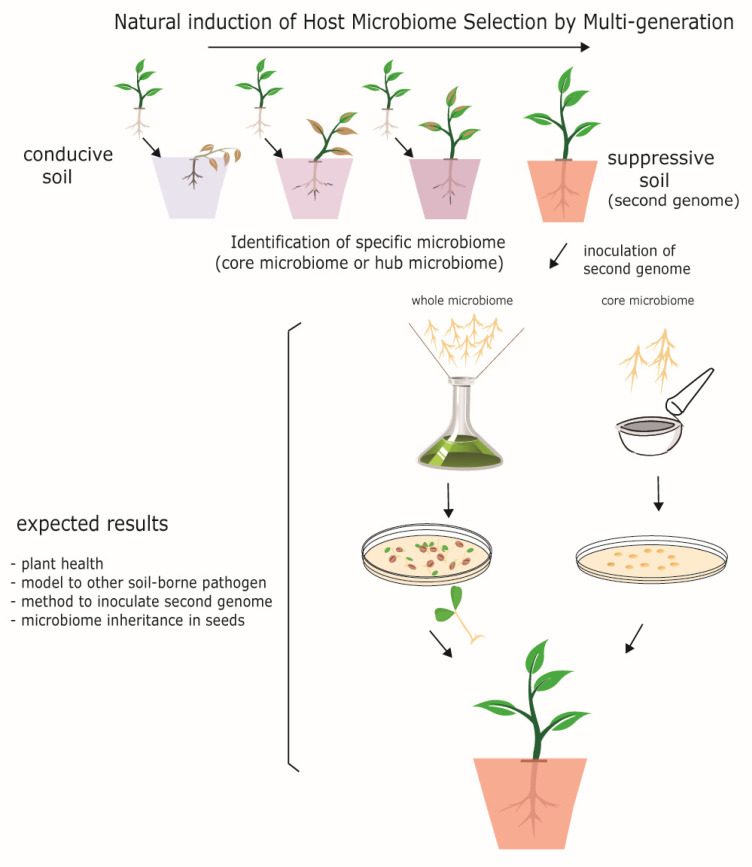
Engineering microbiome by host-mediated microbiome selection to plant health. The natural selection of microbiota over multiple generations can be induced by continual plant sowing in conducive inoculated soils (horizontal transmission). Using this approach, host plants can be used to recruit and self-identify desirable microorganisms that could later be used as inoculants to transfer functional core microbiota, or whole microbiota, in order to obtain healthy plants for subsequent generations (vertical transmission).

The natural selection of microbiota over multiple generations can be managed indirectly (induced) through the existence of three factors: (1) plant model used, (2) abiotic/biotic stressor or inducers, and (3) the desired microbiome. Thus, in the case of soilborne pathogen, this involves continual sowing of plant genotypes (factor 1) against an infective soilborne pathogen (factor 2) in conducive soil (factor 3) until a soil suppressive is obtained (horizontal transmission). In this manner, key specialized functional microbiota (composed of a functional core microbiome and/or microbial hubs) can be identified, and stable synthetic communities can be assembled in order to mediate plant health. Using this approach, host plants can be used to recruit and self-identify desirable microorganisms that could later be used as inoculants to transfer functional core microbiota, or whole microbiota, in order to obtain healthy plants for subsequent generations (vertical transmission [165], Figure 4). For example, Cooper et al. (2021) recently showed that HMME strategy can be used to improve wheat plant growth under drought stress [165]. A further study induced earlier or later flowering times in *Arabidopsis thaliana* plants using HMME selection [159].

Overall, this strategy for microbiota manipulation, which takes advantage of specialized niches (microbial hubs, keystone, and/or core microbiome) offered by disease-suppressive soils, appears to be a promising approach to improve plant health and opens new research opportunities to optimize microbiota. 

On the other hand, from a practical point of view, the identification of a stable microbial hub or microbial keystone taxa that can tolerate perturbations without affecting the microbial community’s composition and favoring plant health would be extremely useful to improve crop production capacity. Moreover, future studies of its implementation should integrate other environmentally friendly strategies for soilborne disease management, such as the use of crop rotation, soil solarization, and biofumigants, among others, widely reviewed by Panth et al. [166].

Finally, although the selection of “microbial hubs” or a “microbial keystone” through a natural selection of successive plant generations is a promising alternative, the application of advanced modeling techniques to detect key bacteria in humans, which are important for a particular trait such as disease resistance or disease detection, must be taken into consideration to be applied in the plant microbiome. In this sense, a mathematical model such as random forest classification (RFC) coupled with the local interpretable model–agnostic explanation (LIME) toolbox has been effectively applied to detect specific taxa from the intestinal and oral microbiome, which served as a predictor for human diseases [167]. In this same way, for more detailed information about mechanistic mathematical models of the gut microbiota, see the review of Bucci and Xavier [168].

## 8. Conclusions

The understanding of the plant microbiome and rhizobiome over the last decades has allowed for the development of integrated approaches to enhance plant fitness. In this article, we propose that engineering the plant microbiome by host-mediated selection can be used to enhance plant health, taking, as a model, the co-evolutive process that occurs in conducive to suppressive soils (multigenerational generation). Using this model, plants can select their own “personalized” microbiome to counteract the negative effects of a specific soilborne pathogen. The application of this natural technology that manages the plant microbiome to control host health will likely lead to the development of the next generation of bioinoculants. 

## Figures and Tables

**Table 1:** Effect of climate change and plant disease incidence.

Climate Variable	Host	Incidence	Pathogen	Location	Reference
>CO_2_	Wheat	increase increase	*Fusarium* sp. *Septoria tritici*	In vitro (Ireland) In vitro (Ireland)	[55]
	Rice	increase	*Magnaporthe oryzae*	Japan	[56]
	Soybean	decrease	*Peronospora manshurica*	USA	[49]
	Soybean	increase	*Septoria glycines*	USA	
		neutral	*Fusarium virguliforme*	USA	
	Duke forest	decrease	*Phyllosticta minima* *Acer rubrum*	USA	[57]
	Barley	decrease	*Erysiphe graminis*	In vitro (UK)	[58]
>CO_2_ + >T°	Wheat, barley, oat, potato, maize	increase	*Globodera rostochiensis* *Meloidogyne chitwoodi* *Phytophthora infestans*	Finland	[59]
	*Eruca sativa*	increase	*Fusarium oxysporum*	In vitro (Italy)	[60]
>T°	Potato	increase	*Phytophtora infestans* (first half of plant growing season)	UK	[61]
		decrease	*Phytophtora infestans* (second half of plant growing season)	UK	
	Wheat	increase	*Puccinia striformis*	USA, Mexico, Denmark, Eritrea	[53]
	Soybean	increase	*Phakopsora pachyrhizi*	In vitro (USA)	[62]
	Banana bunchy top virus (BBTV)	increase	*Pentalotia nigronervosa*	In vitro (USA)	[63,64]
	*Brasica napus*	increase	*Leptosphaeria maculans*	France	[65]
	Maize	neutral	*Fusarium culmorum*	Germany	[66]
		increase	*Fusarium oxysporum*		
		decrease	*Rhizoctonia solani*		
	Cereals	increase	*Fusarium nivale*	Italy	[67]
	Wheat, barley	increase	*Bipolaris sorokiniana*		
	Several early crops	increase	*Pythium ultimum*		
	Horticultural crops	increase	*Sclerotinia minor*		
	Sunflower, sorghum, maize, cotton, soybean, etc.	increase	*Macrophomina phaseolina*		
	Lettuce	increase	*Sclerotinia sclerotiorum*	In vitro (UK)	[68]
<W_a_	Rice	increase	*Magnaporthe oryzae*	France	[69]
	Tomato	increase	*Ralstonia solanaceum*	In vitro (Japan)	[70]
	Potato	decrease	*Streptomyces turgidiscabies* *Streptomyces europaeiscabiei*	In vitro (Norway)	[71]
	Soybean	increase	*Peronospora manshurica*	USA	[49]
		increase	*Septoria glycines*		
		increase	*Fusarium virguliforme*		

**Table 2:** Microorganisms and abiotic factors involved in soilborne pathogen suppression.

Pathogen	Country/Source Soil	Plant	Suppresser	Reference
Fungi				
*Rhizoctonia solani* *Fusarium* sp.	Brazil/pasture, fallow ground, forest	Common bean	Abiotic (hydrolysis of fluorescein diacetate, CO_2_) Biotic (total microbial activity)	[78]
*Rhizoctonia solani*	Egypt	Sugar beet	Plant growth promoting (PGP yeast), *Candida valida, Rhodotorula glutinis, Trichosporon asahii*)	[79]
*Rhizoctonia solani*	India	Rice	*Pseudomonas* spp.	[80]
*Rhizoctonia solani* *Pythium aphanidermatum, Fusarium oxysporum*	Belgium	Mung bean	PGP rhizobacteria (*Brevibacillus brevis, Bacillus subtilis*)	[81]
*Rhizoctonia solani* *Macrophomina phaseolina* *Fusarium solani*	Pakistan	Tomatoes	PGP rhizobacteria (*Pseudomonas fluorescens*, *Pseudomonas aeruginosa, Bradyrhizobium japonicum*)	[82]
*Rhizoctonia solani*	Germany	Sugar beet	Abiotic (pH) Biotic (*Actinomyces, Bacillus, Pseudomona*)	[83]
*Rhizoctonia solani*	Netherlands	Sugar beet	*Proteobacteria, Firmicutes, Actinobacteria*	[84]
*Fusarium* sp.	substrate	Cucumber	Sludge compost: sewage sludge (pig manure), sawdust, matured sludge compost	[85]
*Fusarium* sp.	substrate	Chrysanthemum	Composted sewage sludge into the Pinus bark-based substrate	[86]
*Fusarium* sp.	substrate	Tomatoes	Sewage sludge and yard wastes	[87]
*Fusarium* spp.	China	Peanut	Intercropping of peanut with *Atractylodes lancea*	[88]
*Pythium ultimum*	Sweden	Wheat	Permanent soil cover and a balanced nutrient	[89]
*Fusarium oxysporum*	Algeria	Palm groves	Soil abiotic factors (i.e., clay addition to sansy soil)	[90]
*Fusarium oxysporum*	Korea	Strawberry	Actinobacteria	[91]
*Fusarium oxysporum*	Brasil	Common bean	Pseudomonadaceae, bacillaceae, solibacteraceae and cytophagaceae	[19]
*Fusarium solani*	Pakistan	Tomatoes	PGP rhizobacteria (*Pseudomonas fluorescens, Pseudomonas aeruginosa, Bradyrhizobium japonicum*)	[82]
*Gaeumannomyces graminis*	Chile	Wheat	Soil microbiome	[15]
*Gaeumannomyces graminis*	Chile	Wheat	Endophytic microbiome	[17,22]
*Gaeumannomyces graminis*	Australia	Wheat	Stubble retention and reduced tillage	[92]
Bacteria				
*Ralstonia solanacearum*	Japan	Tomato	Soil bacteria	[93]
*Xanthomonas oryzae*	India	Rice	*Pseudomonas* spp.	[80]
*Streptomyces* spp.	USA	Potato	*Lysobacter*, acidobacteria	[94]
Nematode				
*Heterodera avenae*	UK	Oat	*Verticillium chlamydosporium*, *Nematophthora gynophila*	[95]
*Meloidogyne javanica*	Belgium	Mung bean	PGP rhizobacteria (*Brevibacillus brevis*, *Bacillus subtilis*)	[81]
*Meloidogyne javanica*	Pakistan	Tomatoes	PGP rhizobacteria (*Pseudomonas fluorescens*, *Pseudomonas aeruginosa, Bradyrhizobium japonicum*)	[82]
